# Prognostic Assessment of Diabetics Using Myocardial Perfusion Imaging: Diabetes Mellitus is Still a Coronary Artery Disease Equivalent

**DOI:** 10.2174/1874192401711010076

**Published:** 2017-08-11

**Authors:** Andrea De Lorenzo, Victor F. Souza, Leticia Glerian, Ronaldo SL Lima

**Affiliations:** 1 Clinica de Diagnostico por Imagem, Rio de Janeiro, Brazil; 2 Universidade Federal do Rio de Janeiro, Rio de Janeiro, Brazil

**Keywords:** Diabetes, Coronary artery disease, Aymptomatic, Myocardial ischemia, Myocardial perfusion scintigraphy, Prognosis

## Abstract

**Background:**

Even though diabetes mellitus (DM) has been considered a “Coronary Artery Disease (CAD) equivalent”, that is still controversial, especially in a contemporary population subject to optimized treatment.

**Objective:**

We aimed to assess the cardiovascular risk of diabetics by myocardial perfusion scintigraphy (MPS).

**Methods:**

Consecutive patients who underwent MPS from 2008 to 2012 were studied. Perfusion scores were calculated, and abnormal MPS was defined as a summed stress score >3. Patients were followed for 3±1 years for all-cause death, which was compared between patients with DM (without known CAD) and patients with known CAD but without DM.

**Results:**

Among 3409 patients, 471 (13.8%) were diabetics without known CAD (DM group) and 638 (18.7%) had CAD without diabetes (CAD group). Annualized death rates were not significantly different between DM or CAD patients (0.9 *vs* 1.5%, p=0.09). With normal MPS, death rates were 0.7% for DM and 0.6% for CAD (p=0.8). With abnormal MPS, death rates increased similarly in the DM and CAD groups.

**Conclusions:**

In diabetic patients without known CAD, the rate of death was not significantly different from patients with prior CAD and without DM. Abnormal MPS increased risk similarly in diabetic patients and in those with CAD. These findings suggest that DM may still be considered a high-risk condition, comparable to known CAD, and effectively stratified by MPS.

## 1 INTRODUCTION

Diabetes mellitus (DM) and coronary artery disease (CAD) are closely associated, as diabetics have a high prevalence of CAD and the latter is the leading cause of death in that patient population [1-5]. In 1998, a landmark study demonstrated that diabetics without a history of CAD had approximately the same the risk of future myocardial infarction (MI) as did patients with prior MI but no DM [6], launching the concept of DM as a “CAD equivalent”. This concept thereafter gained wide recognition and served as a meter for establishing several treatment goals for diabetics(*e.g.* lipid levels) [7]. However, this automatic equivalence is not consensually accepted, as other studies have reported differences in risk levels among diabetic patients, according to the presence of coexisting cardiovascular (CV) risk factors [8, 9]. Also, since 1998, intensive research has focused at reducing CV risk in diabetic patients, with various hypoglycemic medications, lipid-control approaches and other interventions [10, 11]. Diagnostic tools have also evolved. More specifically, myocardial perfusion scintigraphy (MPS) has undergone hardware and software improvements which have determined high-quality images with reduced tracer doses and scan times [12, 13].

After over 15 years of patient management and technology changes, the current status of CV risk in diabetic patients and the role of MPS in their risk-stratification remain to be appraised. We therefore sought to assess the prognosis of a contemporary cohort of diabetic patients without known CAD *vs* non-diabetic patients with CAD, trying to re-evaluate the “CAD equivalence” concept, as well as the role of MPS in their risk-stratification.

## 2 METHODS

Our study population consisted of consecutive patients who underwent MPS at a single outpatient Nuclear Cardiology laboratory in Rio de Janeiro, Brazil, and were part of an ongoing prospective registry started in 2008. Patients with non-ischemic cardiomyopathies or significant valve disease were not included in the study database. Up to 2012, 3570 patients were included; they were followed-up by annual telephone interviews until 2014. Among these, 4.5% were lost to follow-up, leaving 3409 for analysis. Patients were divided into 4 groups: patients without DM or known CAD (59.8%), diabetic patients without known CAD (DM group: 13.8%), patients with CAD but without DM (CAD group: 18.7%) and diabetic patients with CAD (7.7%). Known CAD was defined as a history of MI, coronary artery bypass surgery, percutaneous coronary intervention or obstructive epicardial coronary disease (detected by cardiac computed tomographic angiography or coronary angiography) under medical treatment.

All patients signed a written informed consent. Enrolled patients completed a questionnaire with clinical information. Chest pain was classified according to location, precipitants and relief with rest or nitroglycerin [14]. Hypertension, hypercholesterolemia, and DM were defined on the basis of history and/or taking antihypertensive, lipid-lowering or hypoglycemic medications, respectively.

Patients underwent treadmill exercise or pharmacological stress using either dipyridamole or dobutamine infusion [15]. Those undergoing treadmill exercise were instructed to discontinue beta-blockers and calcium channel blockers 48h before testing, and nitrates 6h before testing, whenever possible. Exercise testing was performed using the symptom-limited Bruce protocol. Heart rate, blood pressure, and electrocardiographic tracings were obtained at rest, at the end of each stress stage, peak stress and for each of 5 min after stress.

Technetium-99m sestamibi single-photon emission computed tomography (SPECT) myocardial perfusion scintigraphy was performed using 10-15 mCi for rest and stress phases of a 2-day protocol. For exercise and dobutamine studies, the radioisotope was injected at near maximal stress and imaging was begun 15 to 30 min after testing. For dipyridamole testing, technetium Tc 99m-sestamibi was injected at 2-4 min after the end of the infusion.

MPS was performed using a 2-head gamma camera (Ventri, GE Healthcare, Milwaukee, USA). Post-stress prone acquisitions were routinely performed in men and, if indicated, in women. Images were processed with Evolution for Cardiac (GE Healthcare, Milwaukee, USA) software.

MPS images were visually interpreted by consensus of 2 experienced observers. Semiquantitative analysis was used to define the severity and extent of perfusion abnormalities and employed a 5 point score (0=normal to 4=absence of detectable tracer uptake) for each of 17 myocardial segments, and summed stress score (SSS), summed rest score (SRS), and summed difference score (SDS) were then generated). Abnormal MPS was defined as a SSS >3. Ischemia was considered as a SDS >1. The SDS was converted to percentage of ischemic myocardium by dividing the score by 68 and then multiplying by 100 (as the maximum score is 68 and it should be multiplied by 100 to obtain the percentage). Severe ischemia was defined as a SDS >10%. Post-stress gated short-axis images were processed using quantitative gated SPECT software (Cedars-Sinai Medical Center, Los Angeles, California, USA) and left ventricular ejection fraction (LVEF) was automatically calculated.

Follow-up was performed by telephone interview. Our primary endpoint was the occurrence of all-cause death during follow-up. We also looked at late myocardial revascularization (performed >60 days after MPS) either by angioplasty or coronary artery bypass surgery.

## 3 STATISTICAL ANALYSIS

Continuous variables were expressed as mean ± standard deviation and compared by the Student´s t test or Mann-Whitney´s test, when appropriate. Categorical variables were expressed as number and percentage and compared by chi-square or Fisher´s exact test. A Cox proportional hazards analysis was used to obtain the risk-adjusted odds ratio of baseline (clinical and scintigraphic) factors predicting all-cause death. All-cause death was compared between patients with DM or CAD using Kaplan-Meier curves, which were compared by a log-rank test. Analyses were performed with SPSS software, version 20.0. A two-sided p-value <0.05 was considered significant.

This work has been carried out according to the principles of the Declaration of Helsinki.

## 4 RESULTS

Since the focus of the study was the comparison between patients with DM and patients with CAD, all results presented thereafter refer to comparisons between these 2 patient groups (data from other patient subgroups are presented in Table (**2**). Comparing DM with CAD Table (**1**), the former were less frequently male, but underwent pharmacologic stress more often. Patients with CAD more often had abnormal MPS, ischemic MPS and severe ischemia, higher perfusion scores and lower LVEF (although still in the normal range). Nevertheless, it should be noted that diabetic patients without a history of CAD also had high rates of abnormalities: 25.9% abnormal MPS and 20.8% ischemia.

CAD, coronary artery disease; DM, diabetes mellitus; LVEF, left ventricular ejection fraction; SSS, summed stress score; SRS, summed rest score; SDS, summed difference score MPI

Over 3±1 years of follow-up, all-cause death occurred in 2.8% of DM and 4.4% of CAD patients, or annualized rates of 0.9 *vs* 1.5% (p=0.09). Annualized death rates according to MPS result (normal or abnormal) are shown in Fig. (**1**), and were not significantly different among patients from DM or CAD groups. Importantly, either in patients with DM or CAD, a normal MPS study was associated with <1% all-cause deaths/year (0.7% for DM and 0.6% for CAD (p=0.8). Myocardial revascularization occurred in 10.4% and 16.6% of the patients, respectively (p=0.05).

**Fig. (2):**
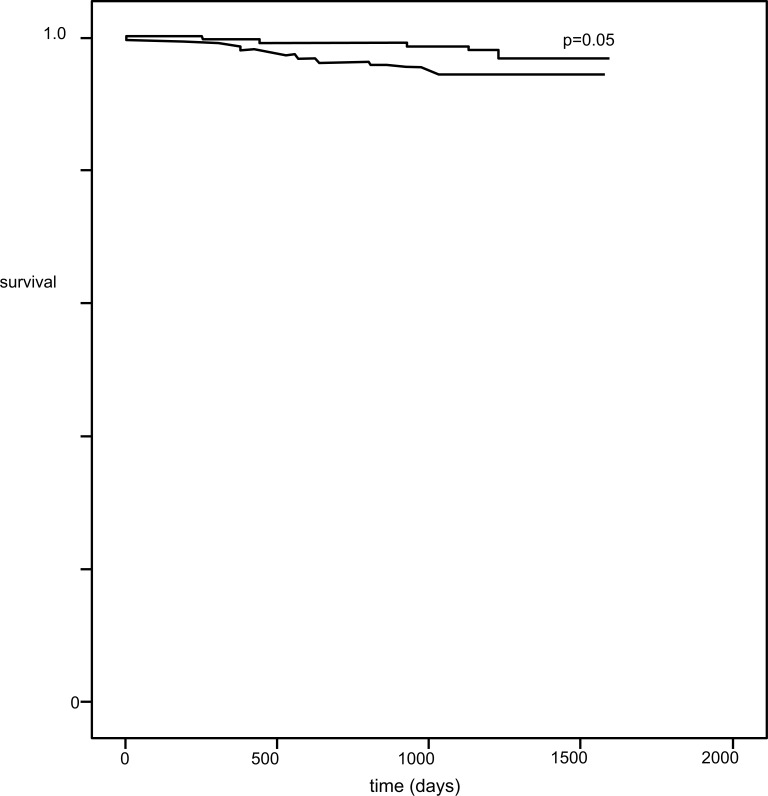
Kaplan-Meier curves for survival free of all-cause death among patients with diabetes (solid line) or known coronary artery disease (dashed line).

In the Cox proportional hazards analysis, SSS was a significant predictor of death (odds ratio= 1.05, p=0.009), as well as the use of pharmacologic stress (odds ratio= 0.36, p=0.01), while DM did not appear as a significant predictor of death (odds ratio=0.91, p=0.7) and CAD had borderline significance (odds ratio=0.52, p=0.05). Kaplan-Meier survival curves displayed a small, nonsignificant difference in event-free survival between patients with DM or CAD (Fig. **2**).

## 5 DISCUSSION

DM and CAD have a strong association, such that since 1998 DM has been considered by many as a “CAD equivalent” [6], a concept which was disseminated and has had important consequences, such as adjustments in CV risk factor goals, clinical practice guidelines *etc*. Despite that, the “CAD equivalence” of DM is a controversial issue, which has been put in question since some studies have not yielded evidence supporting it [8, 9, 16]. In fact, the automatic equivalence has not been incorporated into some practice guidelines, which advocate individually tailored approaches towards risk factor control in diabetic patients [17]. The importance of this issue led us to search for additional information.

As expected, MPS abnormalities were more frequent and severe in patients with CAD. However, diabetic patients also had a high prevalence of MPS abnormalities (25.9%). This is similar to older studies which reported high prevalences of CAD in the overall diabetic population as well as in asymptomatic diabetics [3, 18, 19].

Our results showed that all-cause death was not significantly different between patients with DM or known CAD, even though slightly higher in the latter. The highest mortality was found for patients with DM and CAD (Table 3; data from other patient groups was outside the scope of this manuscript). Importantly, late myocardial revascularization (>60 days after the index MPS study) was also not significantly different between patients with DM or those with known CAD, despite the higher prevalence and severity of myocardial ischemia in the latter.

Our results indicate that MPS was able to risk-stratify both patient groups. Annualized death rates in the setting of a normal MPS were <1%, consistent with prior studies in general populations [20-22]. Patients who died had higher perfusion scores than those who survived, also as previously described [21], and no significant differences were found among patients with DM or CAD.

## 6 LIMITATIONS

As all subjects enrolled in this study were ambulatory patients referred to an outpatient clinic, with overall lower cardiac risk, additional evidence obtained from different subgroups of diabetic patients, with diverse baseline risk levels, may help guide better definitions of which subset of diabetic patients may indeed merit “CAD equivalence”. Nevertheless, we believe that our results will stimulate further debate.

## CONCLUSION

The all-cause death rate of diabetic patients without known CAD was not significantly different from that of patients with known CAD but no DM. Abnormal myocardial perfusion imaging is associated with increased all-cause death both in diabetic patients and in patients with CAD. These findings suggest that DM may still be considered a high-risk condition, comparable to known CAD, and effectively stratified by myocardial perfusion imaging.

## Figures and Tables

**Fig. (1):**
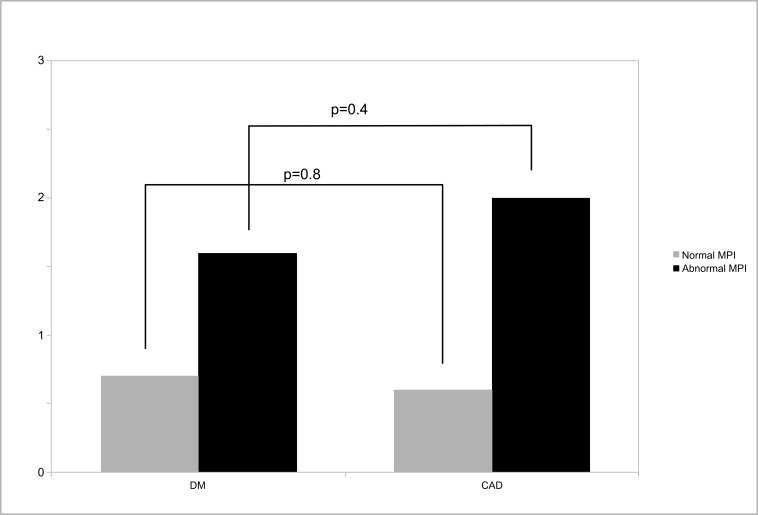
Death rates of diabetics or patients with known coronary artery disease according to myocardial perfusion imaging result

**Table 1:** Baseline data.

	**DM, no CAD (n=471)**	**CAD, no DM (n=618)**
Age (years)	64.0 ± 10.7	65.9 ± 10.7
Male	254 (53.9)	450 (70.5)*
Hypertension	386 (82.0)	362 (56.7)
Hypercholesterolemia	255 (54.1)	335 (52.5)
Smoking	23 (4.9)	38 (6.0)
Asymptomatic	261 (55.4)	383 (60.0)
Typical chest pain	31 (6.6)	64 (10.0)
Prior myocardial infarction	0	296 (46.4)
Pharmacologic stress	241 (51.2)	278 (43.6) †
Abnormal MPI	122 (25.9)	354 (55.5) †
SSS	2.9 ± 4.1	7.0 ± 7.1 †
SRS	1.8 ± 2.6	5.0 ± 6.3 †
SDS	1.2 ± 2.9	1.9 ± 3.2 †
Any ischemia	98 (20.8)	228 (35.7) †
Severe ischemia	24 (5.1)	63 (9.9) †
LVEF (%)	59.8 ± 10.8	52.5 ± 13.3*

numbers are n (%) or mean ± standard deviation

CAD, coronary artery disease; DM, diabetes mellitus; LVEF, left ventricular ejection fraction; SSS, summed stress score; SRS, summed rest score; SDS, summed difference score MPI

* p< 0.05; † p<0.001

**Table 2:** Baseline data from patients not included in the study population (without DM and CAD or with DM and CAD).

	**No DM, no CAD** **(n=2045)**	**DM and CAD** **(n=275)**
Age (years)	60.0 ± 13.0	66.8 ± 9.7
Male	1024 (50.1)	184 (67.0)
Hypertension	1087 (53.2)	222 (80.7)
Hypercholesterolemia	870 (42.5)	166 (60.4)
Smoking	161 (7.9)	17 (6.2)
Asymptomatic	1049 (51.3)	168 (61.5)
Typical chest pain	117 (5.7)	23 (8.4)
Prior myocardial infarction	0	112 (40.7)
Abnormal MPI	321 (15.7)	137 (49.8)
SSS	1.9 ± 3.0	7.0 ± 6.8
SRS	1.3 ± 2.6	5.8 ± 4.4
SDS	0.6 ± 2.0	2.4 ± 3.7
Any ischemia	249 (12.2)	111 (40.4)
Severe ischemia	51 (2.5)	32 (11.6)
LVEF (%)	61.1 ± 19.1	53.6 ± 12.8

numbers are n (%) or mean ± standard deviation

**Table 3:** Annualized death rates of all patient subgroups.

	**no DM, no CAD**	**DM, no CAD**	**CAD, no DM**	**DM and CAD**	**p-Value**
Annualized death rate (%)	0.6	0.9	1.5	2.3	<0.001

p-value for trend across patient categories:

## LIST OF ABBREVIATIONS

CAD: = Coronary Artery Disease
DM: = Diabetes Mellitus
LVEF: = Left Ventricular Ejection Fraction
MPS: = Myocardial Perfusion SPECT
SDS: = Summed Difference Score
SPECT: = Single-photon Emission Computed Tomography
SRS: = Summed Rest Score
SSS: = Summed Stress Score
